# Cloning of the koi herpesvirus (KHV) gene encoding thymidine kinase and its use for a highly sensitive PCR based diagnosis

**DOI:** 10.1186/1471-2180-5-13

**Published:** 2005-03-17

**Authors:** Herve Bercovier, Yolanta Fishman, Ronen Nahary, Sharon Sinai, Amir Zlotkin, Marina Eyngor, Oren Gilad, Avi Eldar, Ronald P Hedrick

**Affiliations:** 1 Institute of Microbiology, Department of Clinical Microbiology, The Hebrew University-Hadassah Medical School, Ein Karen, Jerusalem, Israel; 2 Department of Poultry and Fish Diseases, The Kimron Veterinary Institute, Beit Dagan, Israel; 3 Department of Medicine and Epidemiology, School of Veterinary Medicine, University of California, Davis, California 95616, USA; 4 Department of Infectious diseases, Sheba medical center, Tel Hashomer, 52621, Israel

## Abstract

**Background:**

Outbreaks with mass mortality among common carp *Cyprinus carpio carpio *and koi *Cyprinus carpio koi *have occurred worldwide since 1998. The herpes-like virus isolated from diseased fish is different from *Herpesvirus cyprini *and channel catfish virus and was accordingly designated *koi herpesvirus *(KHV). Diagnosis of KHV infection based on viral isolation and current PCR assays has a limited sensitivity and therefore new tools for the diagnosis of KHV infections are necessary.

**Results:**

A robust and sensitive PCR assay based on a defined gene sequence of KHV was developed to improve the diagnosis of KHV infection. From a KHV genomic library, a hypothetical thymidine kinase gene (TK) was identified, subcloned and expressed as a recombinant protein. Preliminary characterization of the recombinant TK showed that it has a kinase activity using dTTP but not dCTP as a substrate. A PCR assay based on primers selected from the defined DNA sequence of the TK gene was developed and resulted in a 409 bp amplified fragment. The TK based PCR assay did not amplify the DNAs of other fish herpesviruses such as *Herpesvirus cyprini *(CHV) and the channel catfish virus (CCV). The TK based PCR assay was specific for the detection of KHV and was able to detect as little as 10 fentograms of KHV DNA corresponding to 30 virions. The TK based PCR was compared to previously described PCR assays and to viral culture in diseased fish and was shown to be the most sensitive method of diagnosis of KHV infection.

**Conclusion:**

The TK based PCR assay developed in this work was shown to be specific for the detection of KHV. The TK based PCR assay was more sensitive for the detection of KHV than previously described PCR assays; it was as sensitive as virus isolation which is the golden standard method for KHV diagnosis and was able to detect as little as 10 fentograms of KHV DNA corresponding to 30 virions.

## Background

Common carp *Cyprinus carpio carpio *is the most widely cultivated fish for human consumption mainly in Asian and in European countries including Israel [1]. The subspecies *Cyprinus carpio koi *or koi is cultivated as an expensive pet fish especially in Japan but also worldwide [2]. Since 1998, mass mortality among common carp and koi has occurred in the U.S.A, Israel, Germany, England, Italy, Netherlands, and more recently in Indonesia and Japan [3-8]. These outbreaks were due to a deadly infection with a newly recognized virus, the *koi herpesvirus *(KHV) [5]. Intensive fish culture, koi shows, domestic and international trading in the absence of health certifications or inspections have contributed to the rapid global spread of KHV [9]. Initial characterization of the virus showed that KHV resembles members of the family *Herpesviridae *although the virus was clearly different from two previously described herpes-like viruses from fish, *Herpesvirus cyprini *(CHV) and the channel catfish virus (CCV) [5]. Further characterization, including taxonomic relationships of KHV to other viruses will be dependent on more complete genome analyses. The definitive diagnosis of KHV is based on the isolation of the virus from infected fish and is time consuming. Therefore a polymerase chain reaction (PCR) assays to specifically detect the virus in fish tissues was developed by Gilad *et al*. and Gray *et al*. [10,11] and currently these tests are used to complement or even replace virus isolation as a means to detect KHV infections in koi and common carp. The PCR assays based on KHV DNA sequences fortuitously chosen and with no defined function [10,11] are more prone to lose their diagnostic value due to random mutations or deletions than a PCR assay based on sequences of essential virulence or structural genes. We identified from a genomic KHV DNA library an open reading frame (ORF) that encodes a protein with significant homology to thymidine kinase (TK) and developed a new PCR assay based on these sequences that is shown here to be significantly more sensitive than the previously described PCR assays.

## Results and discussion

### Characterization of the KHV TK

**Figure 1:**
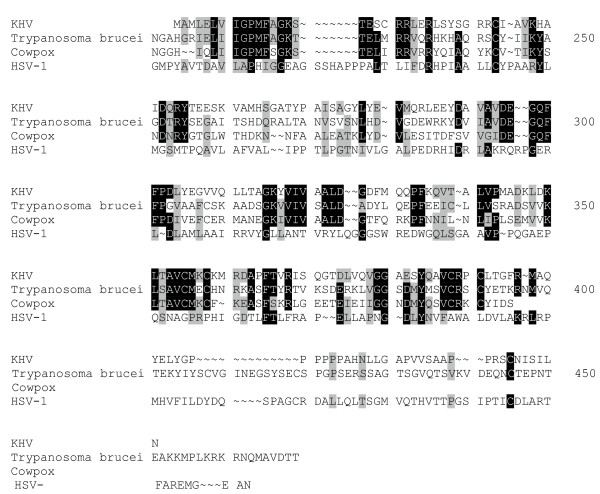
**Amino acids sequence alignment of the KHV TK gene**. Multiple sequence alignment of KHV TK with representative TK sequences from *Poxviridae*, *Herpesviridae *and *Trypanosomatidae *families, performed by the Clastall W algorithm.

**Figure 2:**
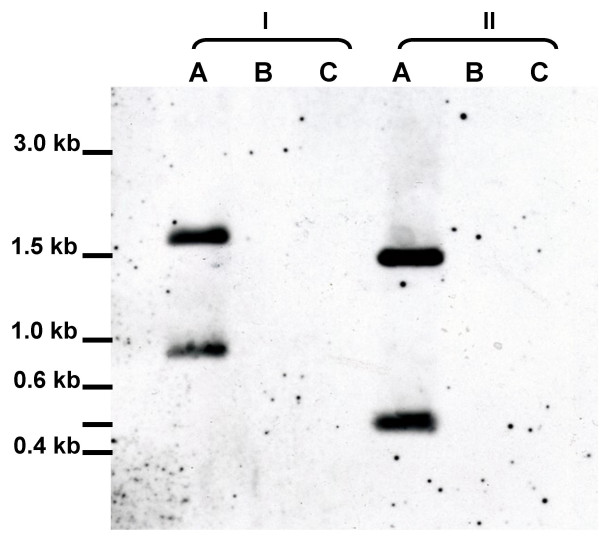
**Southern blot hybridization of Carp and KHV DNA with the TK gene**. KHV (A) and Carp (B) DNA were digested with *Bsa*AI (I) or *Pvu*II (II) and hybridized with a labeled TK probe. BCG (Bacillus Calmette Guerin) DNA (C) was used as a negative control.

When analyzing the sequence of the clones of the genomic KHV library, we identified a hypothetical thymidine kinase gene (AJ535112). At the DNA level, a low homology (Expect = 6^e-05^) was found only with the TK of *Leishmania major *Friedlin (chromosome 21 cosmid L6294) (AL354533). However, when translating the DNA sequence into deduced amino acid sequences, similarities were found between the hypothetical KHV TK and the *Trypanosoma brucei *thymidine kinase (AF395663), the TK of *Leishmania major *(AL354533), and the TKs of *Poxviridae *(AF163844 and U94848) (Figure 1). We could not find any significant similarity between KHV TK and the equivalent genes in any described fish viruses. Southern blot hybridization with the labelled TK gene showed definitively that the KHV TK gene hybridized only with the DNA of KHV and not with carp DNA (Figure 2).

The KHV TK gene had an open reading frame of 216 amino acids encoding a polypeptide with a putative molecular mass of 23.7 kDa. The similarity to eukaryotic TK may reflect a gene acquired from the host [12,13]. The proper nomenclature of the KHV will be solved by a complete genome analysis and not by the analysis of a single gene like in this study or by the histopathology performed on diseased fish [6].

**Figure 3:**
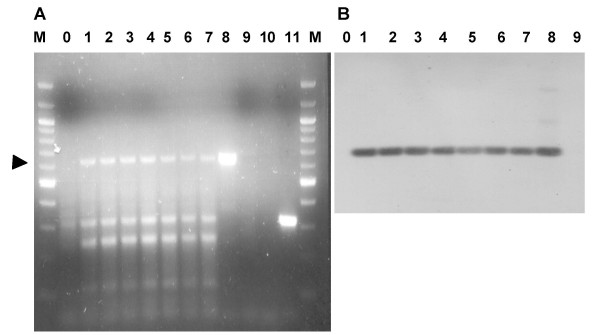
**Analysis of KHV TK mRNA in KF-1 cell line infected with KHV**. KF-1 cells were infected with KHV. At 0 to 7 days post infection total mRNA was prepared from the infected cells and was used in an RT-PCR reaction using primers TK-Rtforward- TK and RTr long (A). 0 time control mRNA was prepared immediately after infection. lanes: M- size markers, 1–8- mRNA of days 0–7 post infection, 9- direct PCR of KHV DNA, 10- uninfected KF-1 cells 11- RNase treated mRNA of day 5 post infection. To confirm the identity of the RT-PCR products, Southern blot hybridization (B) was performed using the cloned TK gene as probe and mRNA of days 0–7 lanes 1–8 consecutively, control KHV – lane 9, and uninfected KF-1 cells as negative control – lane10. The arrow marks the position of the 621 bp product.

The KHV TK gene was expressed *in vivo *in experimentally infected KF-1 cells as shown by RT-PCR analysis. The mRNA of the TK gene was not detected during the first 7 hours of infection (data not shown) but was detected for at least 7 days after infection (Figure 3A). By Southern hybridization, we proved that the fragment amplified by RT-PCR was indeed the TK KHV gene (Figure 3B).

The KHV TK gene cloned into plasmid pYB1 was over expressed in *E. coli *ER2566. SDS-PAGE analysis of the recombinant TK showed a unique band of about 24 kDa, in agreement with the size predicted from the DNA sequence (23.7 kDa) (data not shown). Enzyme activity was determined using tritium labeled deoxy thymidine and deoxy cytidine as substrates. [14]. Preliminary analyses show that the enzyme is specifically active using methyl-^3^H Thymidine (dThd) but had no activity on deoxy (5-^3^H)cytidine (dCyd) similar to other herpesvirus TK. Acyclovir (Samchully Pharmaceuticals South Korea) and Gancyclovir (Hoffmann La Roche Basel Switzerland) in up to 100 molar excess over dThd, did not compete at all with the substrate.

### TK based PCR assay

TK has been shown to be essential for virulence in herpes viruses [15] and therefore is a good candidate for the development of a PCR assay. The KHV infection is an emerging viral disease for common carp and koi. The highly contagious nature of this disease will unfortunately continue to negatively impact the aquaculture industry by increasing the mortality and restrictions on product movements. Rapid diagnosis of the virus is therefore essential for the control of KHV outbreaks. Currently, KHV outbreaks are assessed by multifactorial analyses comprising the *post mortem *observation of gross pathology lesions, the dynamics of mortality in all sizes of fish, the water temperature and the continuing mortality despite chemotherapy. The gold standard method for the diagnosis of KHV infection is based on virus isolation using KF-1 cells followed by PCR testing of the virus isolate [10]. This protocol is time consuming and therefore PCR assays alone have been suggested as possible substitutes for diagnosis of KHV disease [10,11,16,17].

**Figure 4:**
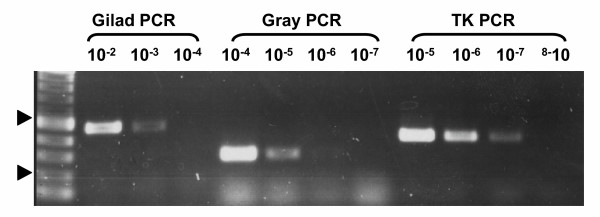
**Sensitivity of three PCR assays for the diagnosis of KHV infection**. A TK based PCR was used to detect KHV DNA and was compared to two other PCR protocols: the Gilad *et al*. (2002) and Gray *et al*. (2002). Details of primers, products and protocols are described in table 1. To compare the sensitivities of the assays, 10 fold serial dilutions of purified KHV DNA were prepared starting from 10 ng DNA per PCR reaction. PCR products were analyzed on a 1% agarose gel and visualized by ethidium bromide. For each protocol the highest dilutions that still give products are shown. The arrows denote the 200 and 500 bp. size markers.

Based on the TK sequence a newly developed PCR assay using the primers, KHV-TKf (5'-GGGTTACCTGTAC GAG-3') and KHV-TKr (5'-CACCCAGTAGATTA TGC-3') has been developed. Under optimal conditions (see Methods) this PCR assay amplified a 409 bp fragment when KHV DNA was used as template (Figure 4). This assay was specific for KHV and did not amplify any fragment when using CCV, CHV or KF-1 cell line DNAs as DNA templates (data not shown). A comparison of the sensitivity of two published PCR assays to the TK PCR assay (Table 1) using for each assay the conditions described by the authors Gilad *et al. *[10] and Gray *et al. *[11] are shown in Figure 4 and Table 2. Using purified KHV DNA as template, the TK PCR assay, whose product was visualized by agarose gel electrophoresis, ethidium bromide staining and UV illumination, was able to detect as little as 10 fg of KHV DNA (Figure 4). Preliminary unpublished data showing that the genome size of KHV is around 290 kb (R. Nahary and H. Bercovier, unpublished data) is in accordance with that estimated by Ronen *et al. *[18] indicate that the 10 fg detected by the TK PCR assay correspond to approximately 30 virions. The two previously described PCR assays were consistently 10 to 1000 times less sensitive (Figure 4) than the new TK PCR. Similar results were obtained when DNA extracted from kidneys of diseased fish were tested by the three PCR assays (data not shown). Recently, real time PCR assay [16] and LAMP assay [17] have been developed for the detection of KHV DNA. The sensitivity of these two methods was not shown to be much higher than that obtained by the TK based direct PCR assay developed in this work.

In addition to assessments of the specificity and sensitivity of the PCR assays, the new assay was compared to virus isolation from koi with different histories in relation to KHV infection. Koi were examined from ponds with active disease, or that had been voluntarily exposed to a KHV challenge and that were with or without disease signs at different times after the experimental exposure. Lastly, populations of koi surviving KHV in Israel were also examined. The survivor groups were the result of a procedure of exposing fish to experimental KHV infection at different seasons of the year and different water temperatures [18] with the claim that they developed resistance to KHV challenge and were free of virus (Table 2). The presence of KHV virus or KHV DNA in the kidney tissues of freshly collected koi was determined with the three PCR assays and the virus isolation method. The results showed that only the TK based PCR assay paralleled the virus isolation method (7 samples positive with both techniques and 6 samples negative with both) proving its sensitivity and its usefulness (Table 2). The two other PCR assays were similar to each other in their performances and did not produce any false positive results but resulted in false negative results (5 false negative) when compared to TK based PCR assay or virus culture. We were able to detect KHV and its DNA in fish that were intentionally infected even two months after the experimental infection (Table 2). These data match with the results obtained by Gilad *et al. *[16] using a Taqman PCR assay to detect the presence of KHV DNA in experimentally infected koi and raise the question of potential carriers in fish exposed to KHV in order to obtain immune survivors.

The kidney was selected as a representative organ for virus and DNA detection because it was shown previously that KHV is found in this organ [10,11,16]. Aliquots of three kidney samples that were positive with the three PCR assays were frozen and were shown also to be positive after 3 months storage at -70°C using the three PCR assays. However the TK based PCR assay was always able to detect viral DNA in the positive samples at a 10 to 100 higher dilution than the two other PCR assays (data not shown) confirming by this its sensitivity and robustness.

These first field evaluations show the advantage of the TK based PCR for detection of KHV DNA as compared to two previously described PCR assays. The TK PCR assay is based on an essential virulence gene that has little chance to be deleted and is therefore potentially more stable/robust than the published PCR assays based on KHV sequences of no defined function [10,11]. These initial trials show that the three PCR assays do indeed give different sensitivities with TK-PCR clearly performing the best. It was more sensitive, and unlike the other assays was able to detect viral DNA in fish samples even two months after experimental exposure to KHV. KHV DNA was not detected among koi two years post experimental exposure if survivors were not held in an infected environment. The carrier status of the survivor population remains unclear. Negative results by PCR may or may not indicate freedom of KHV infection or a viral load lower than the detection threshold. In conclusion, in all cases in our study, the TK PCR was the only direct PCR assay that always paralleled the gold standard viral isolation method. As the PCR assay is easier and less time consuming, this newly developed TK PCR assay is an excellent candidate for a simple and rapid diagnosis of KHV infection.

## Conclusion

The TK based PCR assay developed in this work was shown to be specific for the detection of KHV. The TK based PCR assay was as sensitive as virus isolation which is the golden standard method for KHV diagnosis, and was able to detect as little as 10 fentograms of KHV DNA corresponding to 30 virion particles. It was found to be 10 to 1000 times more sensitive for the detection of KHV than previously described PCR assays. Although Real Time PCR and LAMP technologies may be as sensitive if not more, than the direct PCR assay developed in this study, they have not been yet tested in field conditions and they require, at least for the real time PCR method, more sophisticated equipment and more expensive reagents than the direct PCR.

## Methods

### Viral strains, virus isolation and DNA extraction

KHV-I originating from koi in Israel [10] was propagated on the koi fin cell line (KF-1) which supports the growth of KHV as described by Hedrick *et al*. [5]. Cells were incubated at 20°C. The channel catfish herpesvirus (CCV) strain CA80-5 served as the unrelated herpes-like virus control and was propagated on channel catfish ovary (CCO) cell line. The CCO cell line was grown in minimum essential media (MEM) supplemented with 7.5% fetal bovine serum (FBS), 50 IU penicillin/ml, 50 μg streptomycin/ml, and 2 mM L-glutamine (MEM-7.5). The FBS concentrations of the growth medium were reduced to 2 % (MEM-2) when CCO cells were infected with CCV. Cells were incubated at 25°C following inoculation with CCV.

The CHV DNA was a gift from Dr. T. Sano and Dr. H. Fukuda, Tokyo University of Fisheries [19]. Viral and cell DNAs were extracted according to Gilad *et al*. [10].

KHV was isolated from koi head kidney tissues of infected or diseased fish according to the method described by Hedrick *et al. *[5] with a slight modification. Replicate wells of a 12-well tissue culture plate containing monolayers of KF-1 cells were inoculated with 0.2 ml of a 1:10 dilution (volume /volume or weight/volume) of the original head kidney homogenate. After an adsorption period of 1 h, 2 ml of MEM-2 containing 0.015 M N-2-hydroxyethylpiperazine-N-2-ethane sulfonic acid (HEPES), 50 IU of penicillin/ml, 50 μg streptomycin/ml, and 2 mM L-glutamine were added to each well and the plates were incubated at 20°C. The plates were observed daily for evidence of cytopathic effects (CPE). After 7 days, if there was no CPE, the medium was used to infect new KF-1 cells and incubated as above. Three such blind passages were performed before a clinical sample was described as negative for virus isolation.

### Cloning of the thymidine kinase

KHV genomic DNA fragments following digestion with *Sau*3AI were separated on 1% agarose gels adjacent to a DNA ladder. DNA fragments in the 3–6 kb region were extracted from the gel, purified and ligated overnight at 4°C into a predigested *Bam*HI site of the multiple cloning site arms of the Lambda Zap Express Vector (Stratagene, La Jolla, CA, USA.). The Lambda Zap Express vector allows *in vivo *excision and recircularization of the cloned inserts within the lambda vector to form a phagemid containing the cloned insert. Packaging of the Lambda Zap Express vector and excision of the pBK-CMV phagemid with the inserts from the Lambda Zap-Express vector were done according to the manufacturer's instructions. *E. coli *XLOLR colonies containing the recombinant phagemids were isolated, grown overnight in LB -Kanamycin broth at 37°C and the plasmids were extracted using the Wizard plus SV miniprep kit (Promega, Madison WI, USA).

One hundred clones with an average 3 kb insert were isolated and purified. Sixty clones have been sequenced using primers T3 (5'-AATTAACCCTCACTACTAA GGG-3') and T7 (5'-TAATACGACTCACTATAGGG-3') found on both sides of the *BamH*I insertion site. Inserts were completely sequenced by walking using relevant primers. The sequences of 60 pBK-CMV inserts were compared with the complete virus protein and nucleotide database from the National Center for Biotechnology Information using the BLASTX and BLASTN programs [20] and the Mac Vector software (Oxford Molecular Ltd. San Diego CA. USA).

### Characterization of the recombinant thymidine kinase

The hypothetical TK gene was amplified by PCR from purified genomic KHV DNA using the primers: IMPTK5 (5'-GTTGTTCATATGGCTATGCTGGAACTGGTG-3') and IMPTK3 (5'-GTTGTTTGCTCTTCCGCACAGGATAGATATGTTACAAGAA CG-3'). To prove that the sequences of the amplified TK originated from KHV DNA and not from a contaminating agent or fish cellular sequences, Southern blot analyses were performed. The TK gene was labeled with digoxigenin (DIG) (Roche Applied Science, Mannheim Germany) according to the manufacturer instructions. Purified TK DNA and Carp DNA (1–2 μg) were incubated with 10 U of *BsaA1 *or with *Pvu*II that cut only once within the TK sequence for 4 h at 37°C. DNA fragments were separated by electrophoresis on 1.0% agarose gels and observed after staining with 1% ethidium bromide. DNA was transferred to positively charged nylon membranes (Roche Applied Science, Mannheim Germany) and treated as recommended by the manufacturer (Roche Applied Science, Mannheim Germany). Prehybridization was performed in 10 ml DIG easy hyb solution (Roche Applied Science, Mannheim Germany) within a roller bottle at 65°C for 30 min. Hybridization was carried out in 6 ml of DIG easy hyb containing 250 ng of DIG labeled probe at 65°C overnight. Membranes were washed twice with low stringency wash buffer (2 × SSC+0.1% SDS) at room temperature followed by two washes with high stringency wash buffer (0.1 × SSC +0.1% SDS) at 60°C. The membrane was blocked for 30 minutes with 40 ml blocking solution and then incubated with a 1:10000 dilution of anti Dig AP conjugated antibody (Roche Applied Science, Mannheim Germany). After washing, the membrane was placed on a plastic film, evenly covered with 500 μl of ready-to-use chemiluminescent alkaline phosphatase substrate (CSPD) (Roche Applied Science, Mannheim Germany), and incubated for 5 min. at room temperature. The membranes were then exposed to super RX X-ray film (Fuji photo film, Tokyo, Japan).

The amplified TK gene was cloned into vector pYB1 (Impact system, New England Biolabs, Beverly MA. USA) with an intein tag that allows the purification of the recombinant protein in a non-denaturated form without any extra residues. The TK protein was expressed in *E. coli *ER2566 and purified according to the manufacturer's instructions (New England Biolabs). After affinity purification on a chitin column, SDS-PAGE analysis was performed to characterize the recombinant TK and the enzymatic activity of the recombinant TK was tested.

Thymidine kinase activity was determined by the DE-81 filter paper assay using tritium labeled deoxy thymidine and deoxy cytidine as substrates. [14]. A typical reaction mixture contained in 100 μl of Tris-Cl (140.0 mM) MgCl_2 _(2.0 mM), DTT (1.7 mM), NaF (8.0 mM), PMSF (5.0 mM), ATP (10.0 mM), KHV TK enzyme, and substrates methyl-^3^H thymidine (dThd) (25 ci/mM) or deoxy(5-^3^H)cytidine (dCyd) (22 ci/mM) (Amersham Biosciences, UK). Substrates were used at concentrations between 5–30 mM. Reactions were carried out at 37°C. At 15 seconds intervals, 20 μl samples were removed, and spotted onto the filters. The filters were washed 4 times in 5.0 mM ammonium formate followed by a 15 min wash in Ethanol 95 %. Amount of radioactivity was determined in a scintillation counter (LS 6000TA, Beckman Instruments Fullerton CA. USA).

To demonstrate that the KHV TK is expressed in an experimentally infected cell line, total RNA was extracted using the SV total RNA isolation system (Promega, Madison WI. USA) from KF-1 cells infected with KHV. Uninfected KF-1 cells served as a control. RT-PCR (Access RT-PCR system, Promega, Madison WI. USA) was performed using internal primers of the TK gene (Upstream primer TK-RTf: (5'-ATGGCTATGCTGGAAC-3'); downstream primer TK-RTrl: (5'-TGGAGCGGCT GACACG-3') on experimentally infected KF-1 cells at different time points post infection. PCR products were analyzed on 1% agarose gel. The identity of the amplified products was confirmed by Southern blot hybridization with the labeled KHV TK gene as described above.

### PCR assays

Head kidneys were removed from koi carp infected naturally or intentionally by KHV in order to obtain survivors in Israeli farms [18]. Template DNA from head kidney of koi carp was prepared using High Pure PCR Template Preparation Kit (Roche Diagnostics, Mannheim Germany) according to the manufacturer's recommendations. A TK based PCR assay to detect KHV DNA was developed using specific primers, KHV-TKf (5'-GGGTTACCTGTACGAG-3') and KHV-TKr (5'-CACCCAGTAGA TTATGC-3'), derived from for the thymidine kinase gene (AJ535112) that produced a 409 bp amplicon. The TK based PCR was done using the above primers for 35 cycles of amplification under standard conditions (95°C 5 min, then 35 cycles: 95°C 30 sec, 52°C 30 sec, 72°C 1 min and finally 72°C 10 min).

Two other PCR assays were performed in parallel according to their original description and are denominated Gilad PCR (400 bp product obtained with Primers (5'-GACGACGCCGGAGACCTTGTG-3') and (5'-CACAAGTTCAGTCTGTTC CTCAAC-3') [10] and Gray PCR (290 bp product obtained with primers (5'-GACACCACATCTCAAGGG-3') and (5'-GAC ACATGTTACAATGGTGGC-3') [11] (Table 1).

The specificity of the PCR assays (discrimination of KHV from other viruses) was assessed as described by Gilad *et al. *[10]. A total of 50 ng of DNA obtained from purified KHV-I, CHV, CCV, or uninfected KF-1 cells were used as templates for the PCR assays. The sensitivity of the PCR assays was determined using 10 fold serial dilutions from 10 ng to 0.001 pg of KHV-I DNA and 10 fold serial dilutions of head kidneys (boiled in distilled water for 5 min) from diseased koi carp with proven virus isolation or the extracted DNA. Whole tissue or DNA extracted from negative head kidney samples from uninfected koi carp were run as controls. The resulting amplified products were analyzed by electrophoresis on 1% agarose gels after staining with ethidium bromide.

## Authors' contributions

HB, AE and RPH conceived the study, and participated in its design and coordination and drafted the manuscript. RN, OG, SS and AZ carried out the molecular studies (DNA preparation, PCR development and calibration) and participated in the sequence alignment. YF cloned and expressed the recombinant TK and carried out the enzymatic assays. ME prepared virus cultures and performed PCR assays in diseased fish. All authors read and approved the final manuscript.

## Figures and Tables

**Table 1:** PCR coanditions and primers for the detection of KHV

	Gilad	**Gray**	**TK**
**Primer sequences**	5'-GACGACGCCGGA GACCTTGTG-3' 5'-CACAAGTTCAGTCT GTTCCTCAAC-3'	5'-GACACCACATCTGCAA GGAG-3' 5'-GACACATGTTACAAT GGTGGC-3'	5'-GGGTTACCTGTACGAG-3' 5'-CACCCAGTAGAT TATGC-3'
**Fragment size**	484 bp	290 bp	409 bp
**Enzyme**	1 unit Taq DNA polymerase platinum (Invitrogen)	1 unit Taq DNA polymerase (Promega)	1 unit Taq DNA polymerase platinum (Invitrogen)
**Reaction buffer**	*Tris-HCl 20 nM pH = 8.4 KCl 50 mM MgCl_2 _2 mM (NH_4_)_2_SO_4 _15 mM *Taq platinum buffer	Tris-HCl 60 nM pH = 8.3 MgCl_2 _2 mM (NH_4_)_2_SO_4_15 mM	*Tris-HCl 20 nM pH = 8.4 KCl 50 mM MgCl_2 _1.5 mM *Taq platinum buffer
**Primers / dNTP's **	30 pmole /reaction 400 μM	0.25 μg/reaction 10 μM	0.4 μg/reaction 200 μM
**DNA concentration**	157 ng/reaction)	157 ng/reaction	157 ng/reaction
**PCR reaction volume**	50 μl	50 μl	50 μl
**Program**	95°c for 5 min 94°c for 1 min 68°c for 1 min 72°c for 30 sec 39 cycles 72 C for 7 min	94°c for 1 min 55°c for 2 min 72°c for 3 min 30 cycles 72°c for 7 min	95°c 5 min 95°c 30 sec 52°c 30 sec 72°c 1 min 35 cycles 72°c for 10 min
**PCR apparatus**	Biometra -T Gradient	Biometra -T Gradient	Biometra -T Gradient

**Table 2:** Comparison of three PCR assays and virus isolation from koi carp exposed to KHV

Sample	TK PCR	Gilad PCR	Gray PCR	Virus Isolation	History of the koi carp
1	+	-	-	+	sick fish with clinical symptoms
2	-	-	-	-	survivor (unknown time lap after exposure).
3	-	-	-	-	survivor (unknown time lap after exposure) from a tank with mortality.
4	+	-	-	+	survivor (unknown time lap after exposure) from a tank with mortality.
5	-	-	-	-	survivor moved to 30°C, then to 24°C. no mortality in the tank.
6	+	-	-	+	survivor moved to 30°C, then moved to 24°C. 25% mortality in the tank.
7	-	-	-	-	naïve fish that lived at 16°C. No mortality in the tank.
8	+	+	+	+	fish from tank with mortality.
9	+	-	-	+	fish from tank with mortality.
10	-	-	-	Pooled	survivor, 2 years after experimental infection
11	-	-	-	-	survivor, 2 years after experimental infection
12	-	-	-	Pooled	survivor, 1 year after experimental infection
13	-	-	-	-	survivor, 1 year after experimental infection
14	-	-	-	Pooled	survivor, 2 months after experimental infection
15	-	-	-		survivor, 2 months after experimental infection
16	+	-	-	+	survivor, 2 months after experimental infection
17	+	-	-		survivor, 2 months after experimental infection
18	+	-	-	+	survivor from tank with disease- two weeks post infection.
KF+	+	+	+	+	KF-1 cell line infected with KHV- Positive control.
KF-	-	-	-	-	KF-1 cell line- Negative control.
